# Editorial: Insights in molecular mechanisms of aging 2022

**DOI:** 10.3389/fragi.2023.1339786

**Published:** 2023-12-14

**Authors:** Marco Brotto, Consuelo Borrás

**Affiliations:** ^1^ University of Texas at Arlington, Arlington, TX, United States; ^2^ MiniAging/Freshage Research Group, Department of Physiology, Faculty of Medicine, Centro de Investigación Biomédica en Red Fragilidad y Envejecimiento Saludable-Instituto de Salud Carlos III (CIBERFES-ISCIII), Institute of Health Research-INCLIVA, University of Valencia, Valencia, Spain

**Keywords:** aging, regenerative medicine, skeletal muscle, bone, senescence

We are delighted to announce the successful completion and publication of our second special volume, “*Insights in Molecular Mechanisms of Aging 2022*,” in Frontiers of Aging-Molecular Mechanisms of Aging. We hope that the articles contained within this volume will provide valuable insights into the molecular mechanisms of aging and contribute to the advancement of this field.

We, Dr. Consuelo Borras (University of Valencia, Valencia, Spain) and Dr. Marco Brotto (Bone-Muscle Research Center, University of Texas at Arlington, Arlington, United States) successfully launched this series in 2021 with 5 articles, and 21 authors, reaching 3,900 downloads and 14,000 views.

We just completed the 2022 volume in September of 2023 with 9 articles and 56 authors and we already have 4,154 downloads and over 16,000 views. These numbers offer a glimpse of the great interest in the fast-growing field of the molecular mechanisms of aging.

In chronological order of publication, the following publications have been included in this volume.

Inokuchi et al. contributed with the brief research report “*Age-Dependent Decline in Salinity Tolerance in a Euryhaline Fish*”. These authors addressed the important Research Topic of Euryhaline teleost fish are characterized by their ability to tolerate a wide range of environmental salinities by modifying the function of osmoregulatory cells and tissues, and how aging alters such capacity. They focused their studies on the Mozambique tilapia, *Oreochromis mossambicus*. The authors reported that multiple aspects of osmotic homeostasis, from osmoreception to hormonal and environmental control of osmoregulation, declined in older fish. This decline appears to undermine the ability of older fish to survive and transfer to hyperosmotic environments. As the planet’s climate changes and the acidity, temperature, and salinity of bodies of water change, these studies could also have an important socio-economic impact.

Noh et al. contributed with an original research paper “*G-quadruplexes Stabilization Upregulates CCN1 and Accelerates Aging in Cultured Cerebral Endothelial Cells*”. The premise of these studies was that senescence in the cerebral endothelium has been proposed as a mechanism that can drive dysfunction of the cerebral vasculature, which precedes vascular dementia. Cysteine-rich angiogenic inducer 61 (Cyr61/CCN1) is a matricellular protein secreted by cerebral endothelial cells (CEC). While CCN1 induces senescence in fibroblasts, whether CCN1 contributes to senescence in CEC and how this is regulated was the main goal of their study. Furthermore, they aimed to demonstrate the role of four-stranded Guanine-quadruplexes (G4s) in G-rich motifs of DNA and RNA, since they had previously shown that aging associates with the formation of these complexes. They demonstrated that CCN1 can induce senescence in cultured primary CEC, and provided evidence that G4 stabilization is a novel mechanism regulating the senescence-associated senescence-phenotype component CCN1.

Guzman et al. contributed with an original research article “*Removal of p161INK4 Expressing Cells in Late Life has Moderate Beneficial Effects on Skeletal Muscle Function in Male Mice*”. In this paper, the authors introduced an important rationale for the studies based on the concept that aging results in the progressive accumulation of senescent cells in tissues that display loss of proliferative capacity and acquire a senescence-associated secretory phenotype (SASP). Based on other findings mostly from *in vitro* cell work the tumor suppressor, p16^*INK4A*^, which slows the progression of the cell cycle, is highly expressed in most senescent cells and the removal of p16-expressing cells is beneficial to tissue health; these authors proposed that global p16^*INK4A*^ ablation could have beneficial effects at the whole organismal level at later stages of life. They tested their hypothesis in a model at 20–26 months of age. At 26 months of age, in the mice with the ablation, they found a substantial increase in hind-limb muscle mass and a reduction in inflammatory cytokines. Intriguingly, these beneficial effects were restricted to male mice. These are important studies as they might offer new previously untested therapeutics for sarcopenia. In addition, these studies stress the fundamental importance of scientific studies to address sex differences as a major biological variable.

Inci et al. contributed with a review article *“Translation of Cellular Senescence to Novel Therapeutics: Insights from Alternative Tools and Models*”. The review focused on cellular senescence and senotherapeutic drugs. The review provided a very clear, objective, and concise summary of the molecular mechanisms of cellular senescence (CS) and its role, particularly in the development of cardiovascular diseases (CVD). In the second part of the review, the authors presented a series of research tools including the nonhuman and human models as well as computational techniques for the discovery of novel therapies for CS. In the third part of the review, senotherapeutic approaches that are mainly classified as senolytics and senomorphics were discussed. The article highlighted that although some of these drugs were only discovered 10 years ago, they have already moved to clinical trials, which shows their promise to human health.

Salnikov et al. published a very original research article entitled “*The RNA-seq data analysis shows how ontogenesis defines aging*.” First, the paper presented a global statistical analysis of the RNA-Seq results of the entire *Mus musculus* genome (35,630 genes). The probabilistic hypothesis proposed by the authors for aging was quite intriguing. Aging is a gradual redistribution of limited resources between two major organismal tasks: a) self-sustenance, which depends on the function of the housekeeping gene group (HG), and b) functional differentiation provided by the integrative gene group (IntG). From the 35,630 analyzed, 5,101 belong to the HG group, and 30,529 to the IntG group, and their level of expression had a high level of statistical differences. As the mice matured and reached maturity, the authors detected a shift in the expression levels of the HG group in contrast to the IntG, which provided initial support to their hypothesis. They are now extending their studies to the entire lifespan of the mice. This paper could have a major implication for future aging studies in mice, and this ratio of HG/IntG could perhaps serve as a normalization factor in aging studies or as a biomarker of aging.

Traa et al. published the brief research report “*Endosomal trafficking protein TBC-2 is required for the longevity of long-lived mitochondrial mutants*”, which focused on the effect of disrupting *tbc-2* on lifespan and stress resistance in the long-lived mitochondrial mutants *nuo-6* and *isp-1* in *Caenorhabditis elegans*. The authors argued that since previous studies have shown that the increase in lifespan is dependent on stress-responsive transcription such as DAF-16/FOXO, and the localization of DAF-16 within the cell is dependent on the endosomal trafficking protein TBC-2, it was, therefore, important to determine the role of TBC-2 in this process of life span extension. These authors reported that genetic ablation of *tbc-2* markedly reduced the long lifespans of both mitochondrial mutants, and decreased resistance to chronic oxidative stress in *nuo-6* and *isp-1* mutants but had little or no detrimental effect on resistance to other stressors. They concluded that their data showed the importance of *tbc-2* and endosomal trafficking, while perhaps not accounting for all the effects of the mutation. This study is very important as a model system as well as it utilized the worm *Caenorhabditis elegans.* One interesting possibility is that the response of other stressors that had no effect in the mutants lacking *tbc-2* does not require large sources of energy from mitochondria.

Chaillou and Montiel-Rojas published a mini-review “*Does the blunted stimulation of skeletal muscle protein synthesis by aging in response to mechanical load result from impaired ribosome biogenesis*”? The authors first reminded readers of the crucial roles of skeletal muscles in a host of organismal functions, including metabolic ones. They also pointed out that age-related loss of skeletal muscle mass leads to a reduction of strength, which might develop due to inadequate stimulation of muscle protein synthesis in response to anabolic stimuli (i.e., mechanical load). Their article put forth the hypothesis that ribosome biogenesis is impaired by aging in response to mechanical load, which could contribute to age-related anabolic resistance and progressive muscle atrophy. Authors objectively introduced skeletal muscles, then provided an outstanding overview of the regulation of ribosome biogenesis (see Figure 1 of their paper), next summarized information on ribosomal biogenesis in aged skeletal muscle in response to mechanical load, and finally moved to human studies. They offer a very important overall conclusion that aging appears to impair muscle hypertrophic response to mechanical load in both animals and humans. This impairment in response to load is associated with a deficit in long-term muscle protein synthesis and a blunted increase in RNA concentration. They also concluded that a major contributing factor to these changes with aging is blunted ribosomal biogenesis. This mini-review provides useful information for the aging/sarcopenia and muscle wasting field. It will be important to explore ribosomal-related signaling pathways as possible therapeutics for muscle wasting and weakness in humans.

In the paper entitled “Genetic deletion of KvB2 (AKR6) causes loss of muscle function and increased inflammation in mice” by Manickam et al., the authors investigated the function of the Kvβ2 channel in the regulation of skeletal muscle function during aging by using a mouse model lacking the Kvβ2 channel. They reported a significant reduction in hindlimb skeletal muscle mass and body weight in young Kvβ2 KO mice, which was also significantly reduced in old Kvβ2 KO mice compared with age-matched WT mice. They also found changes in muscle fiber size and distribution. They implemented RNA-seq analysis of gastrocnemius muscles. They found significant increases in genes involved in skeletal muscle development, proliferation and cell fate determination, atrophy, energy metabolism, muscle plasticity, inflammation, and a decrease in circadian core clock genes in young Kvβ2 KO vs. WT mice. These findings bring attention to the important Research Topic of tissue crosstalk. The Kvβ2 channels were thought to be important in cardiac muscle and in this paper, the deletion of Kvβ2 leads to decreased muscle strength and increased inflammation. Since mutations in this channel have been associated with rare cardiac and neuronal diseases, it will be very important to also consider the effects on muscles in these diseases. It will be exciting to see if these studies will also lead to new therapeutic leads.

Awad et al., with their paper entitled “*Revolutionizing bone regeneration: advanced biomaterials for healing compromised bone defects*”. In this review, the authors explored the application of novel biomaterials in craniofacial bone defects with a focus on the aging population. They highlighted that a significant portion of individuals affected by traumatic bone defects in the craniofacial area belong to the aging population. They emphasized the urgent need for innovative biomaterials to address the declining rate of new bone formation associated with age-related changes in the skeletal system. This article comprehensively reviewed the burden and complexity of craniomaxillofacial injuries, the intensive inflammation and elevated oxidative stress associated with aging, the need for antioxidants for bone healing, and current biomaterials and tissue engineering treatment strategies. The authors focused on autografts, allografts, xenografts, and fixative metal-implants and their bioactive coatings.

Further, the review covered the ceramic, polymers, and nano-composite resorbable materials used in bone regeneration. The authors also explored the use of novel semiconductor bioactive coatings as a superior solution for titanium implant surface modification. Overall, this review indicated that the use of improved methods of manufacture and materials with intrinsic properties or the release of small molecules or drugs to target aging mechanisms to regulate cellular aging will be key to improving the outcome for patients and meeting the burden of care.

In summary, this volume sheds light on the progress and challenges in the molecular mechanisms of the aging field and we hope it will inspire students, fellows, and scientists in the aging area of research in years to come.

We congratulate all 56 authors who contributed to this successful volume.

